# Forging biofilms: metal-induced microbial responses in biofilm formation

**DOI:** 10.1128/jb.00247-25

**Published:** 2025-10-16

**Authors:** Sarah L. Price, Eric P. Skaar

**Affiliations:** 1 Department of Pathology, Microbiology, and Immunology, Vanderbilt Institute for Infection, Immunology, and Inflammation, Vanderbilt University Medical Center 204907, Nashville, Tennessee, USA; University of Southern California, Los Angeles, California, USA

**Keywords:** competition, pathogenesis, metals, biofilms

## Abstract

Biofilms are a major contributor to antibiotic resistance and persistence in hospital environments. These bacterial communities form protective structures that shield microbes from various stressors, such as oxidative stress, pH fluctuations, osmotic pressure, and nutrient deprivation. As our understanding of biofilms has grown, it has become evident that metals play a crucial role in several aspects of biofilm biology. Metals are involved in regulating biofilm formation, facilitating communication and competition among bacteria, and supporting the structural integrity and adherence of bacterial cells within the biofilm. This review discusses the complex relationship between metals and biofilms during bacterial pathogenesis to emphasize how the availability of metals influences biofilm dynamics. We explore mechanisms through which metals impact biofilm architecture and resilience, as well as the ability of bacteria to evade host immune defenses and outcompete other microbes. In polymicrobial environments, some bacteria use metals to collaborate with other microbes within the biofilm, whereas others deprive neighboring microbes of essential metals to dominate the biofilm community. Additionally, metals have roles beyond their nutritional value, where they can promote the integrity and stability of biofilms. By understanding these interactions, researchers can gain valuable insights into the significance of metals in biofilm-associated infections. This knowledge can help identify potential therapeutic targets that will lead to the development of new strategies to combat biofilm-related infections and improve patient outcomes.

## INTRODUCTION

Biofilms are complex communities of microorganisms embedded within a self-produced, or host-derived, extracellular matrix (1). The scaffold is rich in polysaccharides, proteins, and nucleic acids to support the structural integrity of the biofilm and help bacteria adhere to surfaces and resist external stressors (1–3). These congregations play a critical role in the pathogenesis of various infectious diseases by enhancing bacterial survival, promoting antibiotic resistance, and facilitating the sustainability and spread of chronic infections (4, 5). The NIH and CDC report that biofilms are responsible for up to 80% of microbial infections, including more than 60% of hospital-acquired (nosocomial) infections (6). Furthermore, biofilm-associated infections are often chronic and recurrent, as bacteria can disperse from the biofilm to establish new sites of infection, complicating treatment and leading to prolonged antibiotic use (7–10). Bacteria living in a biofilm can exhibit up to 1,000-fold more antibiotic resistance than planktonic cells (11). Biofilms can be found in numerous tissues in the body and on prosthetic or implantable devices (12–17). Bacteria colonized in aggregates on abiotic surfaces or in mucosal surfaces in the human host can support the increase in antibiotic resistance (18–21). The development of a greater understanding of biofilm dynamics is crucial for the identification of effective strategies to target and disrupt these resilient bacterial communities.

Biofilms form through free-floating or attached aggregates in host tissues and the bloodstream (22). Bacterial aggregates embed in protective matrices for stability and resilience against environmental stress and the host immune response (23, 24). The extracellular matrix not only acts as a physical barrier but also creates a microenvironment that promotes population-wide communication through quorum sensing that allows bacteria to coordinate their activities, such as gene expression and virulence (25–27). Additionally, biofilms can form complex, multilayered structures with nutrient gradients that support the survival of bacteria even in nutrient-limited conditions, including during metal deprivation (28, 29). This ability to adapt and survive makes biofilms a major contributor to the persistence of infections, particularly in hospital settings where medical devices like catheters, prosthetic implants, and even ventilators outside of the human host can become colonized (30).

A key factor that influences biofilm development and structure is the availability of essential micronutrients, such as metals, which play crucial roles in various biochemical processes vital to bacterial physiology (31, 32). Since metals are critical for bacterial growth and biofilm formation, targeting their availability serves as a promising therapeutic strategy to mitigate infections caused by biofilms. Bacteria utilize substantial resources to acquire metals and maintain metal homeostasis (33). Metals participate in various biochemical processes, including enzymatic reactions, electron transport, and structural stability of cellular components (31). Nutrient metals, such as iron, zinc, and manganese, are particularly influential in bacterial physiology and significantly impact biofilm formation. In bacteria, metals influence processes including bacterial metabolism, virulence factor function, and biofilm generation and are cofactors incorporated into metalloproteins, including metalloenzymes, storage proteins, and transcription factors (32, 34, 35). Because bacteria require nutrient metals, eukaryotic hosts have evolved dedicated mechanisms to sequester and restrict access to these metals to inhibit colonization by microbial invaders, a process termed nutritional immunity (36–40). Nutritional immunity is mediated by a network of metal-binding proteins, including transferrin, lactoferrin, calprotectin, and lipocalin-2, which regulate metal availability in circulation and at mucosal surfaces (41). The distribution, chemical state, and accessibility of these metals vary significantly across tissue types and are further altered during disease, creating distinct metal landscapes that influence microbial colonization and biofilm formation. In healthy tissues, iron is bound extracellularly to transferrin in its ferric form, maintaining low levels of free iron (41). Zinc and manganese are similarly restricted, particularly at sites of inflammation where neutrophils release calprotectin to bind and withhold these metals (42, 43). Lactoferrin is also secreted by neutrophils and sequesters iron (44). This tightly regulated metal environment helps prevent microbial growth and supports immune function. In inflamed or damaged tissues, changes in pH, oxygen tension, and immune cell activity can alter metal speciation and increase local metal concentrations (45). The cystic fibrosis airway favors an increasing ferrous iron pool that supports biofilm development as infections progress (45). Tissue injury and immune dysfunction can also impair the deployment or effectiveness of metal-sequestering proteins, resulting in elevated levels of bioavailable metals (46). In a diabetic wound model, nutritional immunity is unable to control infection, and the bacteria succeed in colonizing the tissue (46). These shifts in metal availability can promote microbial persistence, biofilm formation, and resistance to host defenses.

**Fig 1:**
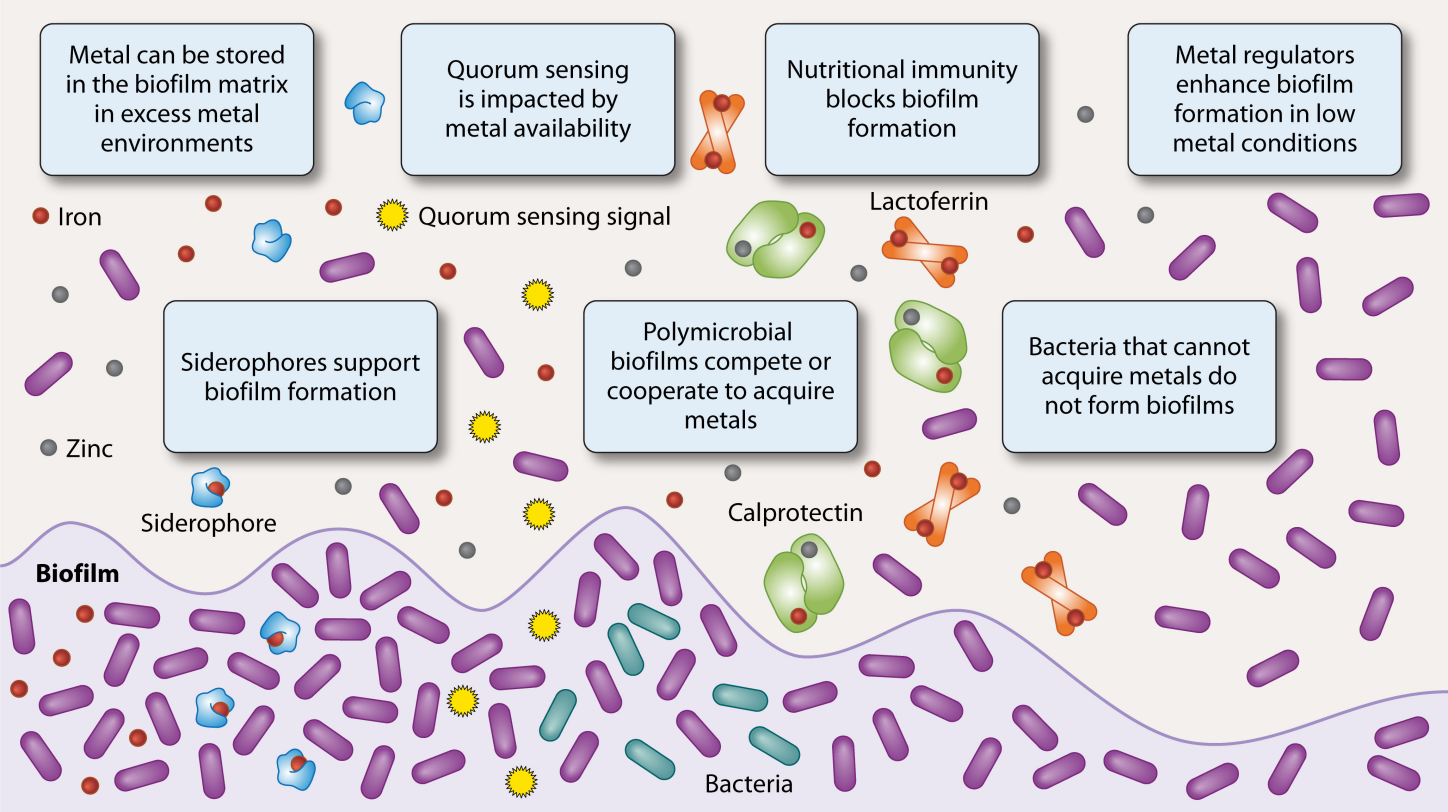
Regulatory themes across metals in biofilm communities. Bacteria within the biofilm matrix are shown interacting with metal ions through various processes: (1) metal storage in the extracellular matrix under high-metal conditions (2); siderophore production to facilitate metal acquisition and support biofilm development (3); quorum sensing modulation by metal availability, affecting intercellular communication (4); polymicrobial interactions, including competition and cooperation for metal resources (5); host-mediated nutritional immunity, with calprotectin and lactoferrin preventing bacterial binding to inhibit biofilm formation; and (6) metal-responsive gene regulation, promoting biofilm formation under metal-limited conditions.

This review explores the multifaceted roles of metals in biofilm formation and bacterial pathogenesis (Fig. 1). The review begins with an analysis of how metal starvation and excess metal conditions regulate biofilm development through metal-responsive transcriptional regulators and stress adaptation mechanisms. It then addresses the influence of metals on quorum sensing, focusing on how iron and manganese modulate bacterial communication and collective behavior. The role of siderophores in biofilm formation is explored, emphasizing their importance in iron acquisition and their emerging functions in microbial competition and host interaction. Polymicrobial biofilm metal interactions are discussed to illustrate how metals shape interspecies dynamics and survival strategies in mixed microbial communities. Clinical examples of nutritional immunity’s impact on biofilm-associated infections are presented, followed by an evaluation of metal-dependent therapeutic strategies aimed at preventing and eradicating biofilms. Insights into these metal-driven processes reveal potential targets for therapeutic intervention and support the development of more effective treatments for persistent infections.

## METAL IN BIOFILM REGULATION

### Metal starvation

In the vertebrate host, bacteria must rapidly respond to immune mechanisms initiated to inhibit bacterial colonization, including metal restriction. In response to nutritional immunity, pathogens have evolved sophisticated mechanisms to acquire these limited metal ions in the vertebrate host environment (37). To maintain metal homeostasis, bacteria encode metal-responsive transcriptional regulators to control metal abundance in the cell and contribute to stress responses. Paradigmatic examples of microbial metalloregulators, including Fur (ferric uptake regulator), Zur (zinc uptake regulator), and Mur (manganese uptake regulator) (47–52). The gram-positive pathogen *Staphylococcus aureus* is an important cause of infection and a model organism for the study of metal metabolism. *S. aureus* can colonize practically every body site and must readily adapt to nutritional immunity in each tissue. *S. aureus* responds to host iron limitation through the production of iron transporters and synthesis and secretion of siderophores, which are small molecules that bind and transport iron into the bacterial cell, transcriptionally controlled by the global regulator Fur (53). During iron restriction, *S. aureus* induces biofilm formation through the release of Fur-dependent gene expression to secrete two adhesion proteins: the extracellular adhesion protein (Eap) and the extracellular matrix protein-binding protein (Emp) (54, 55). Emp and Eap bind to proteins, like fibronectin, fibrinogen, and some forms of collagen (56, 57). Furthermore, the matrix formed by Eap proteins inhibits leukocyte invasion and phagocytosis of bacteria within the *S. aureus* biofilm (58). Eap adhesins also support biofilm formation on prosthetic joint implants (58). To overcome host-imposed metal restriction, *S. aureus* relies on regulatory systems for pathogenesis and persistence across diverse host environments.

*Staphylococcus epidermidis* is an opportunistic pathogen that is typically part of the skin microbiota and can also form biofilms on catheters and surgical implants. *S. epidermidis* is responsible for at least 22% of bloodstream infections in intensive care unit patients in the United States (59). Biofilms are a critical component of virulence for *S. epidermidis*. During infection in the bloodstream, *S. epidermidis* upregulates genes involved in iron uptake and metabolism during biofilm formation (Table 1) (60). As a primary skin colonizer, *S. epidermidis* is impacted by extreme iron conditions during early biofilm development (61). Iron chelation induces extracellular slime production by *S. epidermidis*, which can serve as the matrix of the biofilm (Table 1) (62). Furthermore, biofilm development by the gram-negative opportunistic pathogen *Pseudomonas aeruginosa* is regulated by the repressor Fur through intracellular iron availability (63). In the presence of iron limitation, a *fur* mutant can produce a biofilm structure, whereas wild-type bacteria cannot, suggesting that Fur mediates iron signaling to induce metal acquisition systems for biofilm generation (Table 1) (63). These three examples represent how iron can regulate biofilm adaptations in bacteria.

**TABLE 1:** Metal-dependent mechanisms utilized by bacteria during biofilm processes

Bacteria	Infection site	Metal	Phenotype	References
*Acinetobacter baumannii*	Lung and medical devices	Fe	Fe limitation increases biofilm formation Fe limitation leads to increased secretion of AHL and siderophores to support biofilm formation	(64–66)
*Bacillus anthracis*	Lung, gastrointestinal tract	Zn	Zn-regulated biofilm formation by Zur	(67)
*Bacillus subtilis*	Gastrointestinal tract	Fe	Bacillibactin supports biofilm formation	(68)
*Burkholderia cenocepacia*	Lung	Fe	Loss of glycosylation enzyme decreases biofilm formation and siderophore production	(69)
		Mn	Mn uptake regulates quorum sensing	(70)
*Enterococcus faecalis*	Blood, urinary tract, skin, medical devices	Fe	Fe increases biofilm growth through glycerol metabolism Promotes growth of *E. coli* in a polymicrobial biofilm through enterobactin production in Fe limitation Restricts growth of *P. aeruginosa* by chelating Fe in polymicrobial biofilm	(71–74)
		Mn	Mn-regulation biofilm formation by EfaR	(73, 75, 76)
*Escherichia coli*	Urinary tract	Fe	Yersiniabactin supports biofilms in Fe-limitation	(77)
*Helicobacter pylori*	Stomach	Fe, Zn, Mn, Ni	Calprotectin metal sequestration induces biofilm formation	(78)
*Klebsiella pneumoniae*	Lung, liver	Fe	High Fe concentrations in the liver support biofilm formation	(79, 80)
		Zn	Biofilm increases resistance to Zn intoxication	(81)
*Pseudomonas aeruginosa*	Cornea, lung, wound, bloodstream	Fe	Fe-regulated biofilm formation by Fur	(63, 82–91)
			Osmotic stress enhances Fe uptake and biofilm growth	
			Pyoverdine and pyochelin are important for biofilm formation	
			QS is stimulated during low iron conditions to disperse biofilms	
			The biofilm exopolysaccharide, Psl, is enhanced in high Fe and stores Fe in the matrix	
			Increased Fe availability renders competitive type VI secretion system behavior	
			Pyoverdine secretion chelates Fe from *Aspergillus fumigatus* in the biofilm to reduce biofilm growth	
			Lactoferrin Fe sequestration blocks biofilms	
		Zn	Pyoverdine is important for Zn-dependent biofilm formation	(29)
			Calgranulin Zn chelation reduces biofilm formation	(92)
		Fe, Zn, Mn, Ni	Calprotectin represses biosynthetic genes responsible for production of antimicrobials against *S. aureus* in polymicrobial biofilm	(29, 93, 94)
		Fe, Zn	Siderophores are upregulated in biofilms with *Candida albicans,* and metal limitation impacts the polymicrobial biofilm	(95–98)
*Staphylococcus aureus*	Skin, lung, bloodstream, medical devices	Fe	Fe-regulated biofilm formation by Fur, where Fe limitation leads to the expression of adhesion proteins	(54, 55)
			Converts a siderophore produced by *P. aeruginosa* to an analog with a reduced affinity for Fe to promote a polymicrobial biofilm	(99)
*Staphylococcus epidermidis*	Skin, bloodstream, medical devices	Fe	Fe availability regulates biofilms	(60, 61)
			Low Fe induces matrix production	(62)
			Stimulated by catecholamines to form biofilms by stealing Fe from transferrin	(100, 101)
*Streptococcus mutans*	Oral	Mn, Fe, Zn	Highly upregulates metal transporters to create a harsh biofilm environment	(102–104)
		Zn	Zinc intoxication impairs biofilm formation	(105)
		Cu	Copper intoxication impairs biofilm formation	(106)
*Yersinia pseudotuberculosis*	Gastrointestinal tract	Fe and Zn	Metal-regulated biofilm formation by Fur and Zur	(107, 108)

Zinc is an essential element and serves as an electrophilic catalyst for enzymes and a protein scaffold (109). It is estimated that 5%–6% of bacterial proteomes consist of zinc-binding proteins, emphasizing the need for mechanisms of zinc acquisition in bacteria (110). When *S. aureus* and *P. aeruginosa* encounter zinc restriction *in vitro*, the same stimulus results in an opposite response. *S. aureus* shows an increase in biofilm formation beginning at 24 h at higher concentrations of commercial zinc chelator, although the rate of accumulation decreases (111). Meanwhile, *P. aeruginosa* with the same stimulus results in increased biomass over time but does not show alteration in temporal dynamics (111). On the other hand, the addition of zinc activates intercellular adhesion amongst *S. aureus* cells (112). This occurs through the release of surface protein SasG that mediates cell-to-cell adhesion, which also supports adhesion with *S. epidermidis* (112). Together, these findings illustrate the nuanced and divergent strategies employed by bacteria in response to zinc availability and their pivotal role in modulating biofilm behavior and intercellular interactions.

Zur is a member of the Fur superfamily of transcriptional regulators and manages zinc homeostasis in various bacteria (50). *Yersinia pseudotuberculosis* is a gram-negative pathogen that inhabits the gastrointestinal tract and can spread by lymph circulation to the spleen and liver (113). Zur regulates the highly conserved zinc transporter ZnuABC and the type VI secretion system in *Y. pseudotuberculosis* for zinc uptake (114). In *Y. pseudotuberculosis*, Zur and Fur facilitate biofilm generation in zinc and iron deficiency, respectively (Table 1) (107, 108). In the absence of Zur, biofilm formation is ablated, suggesting that zinc regulation is vital for biofilm development (108). Biofilm formation may be inhibited due to the lack of quorum sensing, since in *Y. pseudotuberculosis,* Zur regulates cell-to-cell communication (108). Furthermore, Zur supports sugar acquisition, glycan biosynthesis, and transmembrane transport that could potentially lead to the production of extracellular polysaccharides to generate the biofilm matrix (108). Biofilm formation is also regulated by Zur in the causative agent of anthrax, *Bacillus anthracis* (Table 1) (67). Zinc homeostasis is essential for bacterial survival and biofilm development in pathogens like *Y. pseudotuberculosis* and *B. anthracis*. By controlling zinc uptake systems, quorum sensing, and metabolic pathways involved in matrix production, Zur plays a central role in coordinating the cellular processes necessary for biofilm formation.

Manganese is a critical modulator of virulence due to its role in enzymes, signal transduction, and protection against oxidative stress. As a keystone pathogen in dental caries, *Streptococcus mutans* resides in biofilms that form on the surfaces of teeth, primarily known as dental plaque (115). For virulence, *S. mutans* modifies biofilm architecture to facilitate an environment for the proliferation of acidogenic and aciduric bacteria and to eliminate beneficial microbes (116, 117). Bacteria in polymicrobial biofilms, such as dental plaque, must also compete with other oral residents for nutrients like metals. During manganese restriction, *S. mutans* highly upregulates two genes, *mntH* and *sloC*, encoding a manganese transporter and a lipoprotein receptor, respectively. Inactivation of these genes decreases manganese uptake and attenuates early biofilm formation in manganese-rich and -limited conditions (102). Furthermore, the lipoprotein SloC is regulated by SloR in response to iron and manganese uptake, and without SloR, *S. mutans* exhibits an altered, aggregated biofilm phenotype (103). Moreover, *S. mutans* requires the zinc transporter AdcABC to scavenge environmental zinc and colonize the tooth surface to form caries (104). Manganese, iron, and zinc support *S. mutans*’ ability to thrive in low pH and oxidative stress, which is critical for the cariogenic potential of the bacterium; thus, lacking these key nutrients impedes biofilm formation (Table 1) (104). Overall, *S. mutans* relies on metal acquisition to form a harsh biofilm environment that allows the bacterium to outcompete other bacteria.

Metals can play a more complex role outside of typical nutritional metabolism in biofilm regulation. During infection of the vertebrate host, bacteria are faced with extreme conditions, such as pH, osmotic, and oxidative stress. For instance, *P. aeruginosa* must survive high osmotic stress in the cystic fibrosis lung environment (118). In *P. aeruginosa*, the BfmRS two-component signaling system responds to osmotic stress. Furthermore, BmfR is activated in iron limitation to induce expression of siderophore genes (Table 1) (119). The two-component signaling system BfmRS is also important for quorum sensing and biofilm formation in *P. aeruginosa* and *Acinetobacter baumannii* (64, 82). *A. baumannii* is a gram-negative opportunistic pathogen that causes life-threatening infections in immunocompromised individuals and can lead to prolonged stays in intensive care, extended antibiotic exposure, and ventilator therapy (120). Similarly, *Pseudomonas putida* is a nosocomial pathogen that can cause bacteremia in immunocompromised individuals (71). *P. putida* increases siderophore production in response to high osmolarity, suggesting that iron uptake is used to combat this stress (121). The mechanism for iron to hinder damage from osmotic pressure remains undefined. Together, biofilm generation and iron influx build a response against osmotic pressure for *P. aeruginosa* survival and represent an unexpected strategy from a signaling system that could represent a therapeutic target.

### Excess metals

Bacteria must acquire metals to maintain their cellular needs, but in excess, these same elements are toxic, can cause mismetalation to proteins and enzymes, and lead to reactive oxygen species production (122–125). The nosocomial pathogen *Enterococcus faecalis* is a ubiquitous member of the gut microbiota and a frequent cause of biofilm-associated infections. *E. faecalis* biofilms are found during endocarditis, urinary tract infections, wound and surgical site infections, and medical device-associated infections. *E. faecalis* adapts across changing iron environments and can withstand high iron concentrations (126–129). Iron increases *E. faecalis* biofilm growth, where the addition of iron does not augment growth in planktonic cells (72). Furthermore, *E. faecalis* stores excess iron in the biofilm matrix and is able to utilize iron to drive glycerol uptake for energy production that stimulates biofilm development (Table 1) (71). This iron-dependent response allows *E. faecalis* to thrive in iron-rich environments, such as the blood and gastrointestinal tract, where the bacterium forms biofilms.

*E. faecalis* requires manganese as an essential cofactor (130). EfaR is a major regulator of manganese transporters in *E. faecalis* (75). Inactivation of *efaR* impairs the ability of *E. faecalis* to form biofilms, suggesting that EfaR regulates systems involved in biofilm formation (Table 1) (75). These data suggest that the manganese regulation factor EfaR is an important modulator of *E. faecalis* virulence, and there is a link between manganese homeostasis and biofilms. In *E. faecalis*, a gene that encodes for a cation efflux transporter, MntE*,* is manganese responsive and utilized to efflux manganese out of the cell when manganese levels are high (73). Furthermore, bacteria lacking MntE show decreased planktonic and biofilm growth with excess manganese (Table 1) (73, 76). However, MntE can also transport iron and magnesium (72, 73). When iron levels accumulate in the bacteria in a *mntE* mutant, glycerol catabolic genes are upregulated to enhance biofilm growth (73). These studies highlight how *E. faecalis* balances metal acquisition and detoxification to support biofilm formation and virulence, with key regulators like EfaR and MntE to coordinate metal homeostasis and energy metabolism in diverse host environments.

Phagocytes can mobilize zinc to intoxicate bacteria during infection. Excess zinc levels prevent bacteria from taking in manganese and increase sensitivity to oxidative stress (131). ZntA is a common zinc efflux pump used by bacteria to overcome zinc intoxication. In *Klebsiella pneumoniae*, which is known to cause both community-acquired and nosocomial infections, ZntA is the primary zinc efflux pump. Without ZntA, biofilm growth is enhanced in *K. pneumoniae* when zinc is in excess (Table 1) (81). Resistance to zinc intoxication is improved by the formation of biofilms, which suggests that components of the biofilm matrix may act to buffer or sequester metal ions (81). In high zinc stress, *S. mutans* lacking the zinc exporter ZccE shows reduced ability to form biofilms (Table 1) (105). This is linked to lower expression of key biofilm-related genes like *gtfB*, *gtfC*, and *nlmC* that are essential for producing the biofilm matrix (105). High concentrations of copper are also utilized by phagocytes to kill bacteria (132). In *S. mutans,* the CopYAZ operon encodes a copper efflux system that maintains copper homeostasis (133). Loss of CopYAZ reduces the transcription of genes involved in biofilm matrix production and biofilm formation in *S. mutans* (Table 1) (106). These findings indicate that zinc and copper detoxification limits *S. mutans* growth in dental biofilms. Overall, bacteria must overcome host-induced metal intoxication through efflux and biofilm formation to resist metal stress and persist during infection.

Bacteria form biofilms across many surfaces in the eukaryotic host. In each biological niche, specific metal availability and concentrations can vary drastically. In metal limitation, bacterial stress responses are triggered, and biofilms are used as a security measure when resources are low. However, if intracellular regulators detect high metal levels, biofilm response, along with metal acquisition genes, is induced. In this case, bacteria can store the metals in the biofilm matrix to be used as an ion shield (134). Although there may be trends in how bacteria adapt to these environments, bacteria must be dynamic in how they regulate and form biofilms to suit the resources that are available to them. Although it is understood that metals contribute to the control of biofilm development and stability, many questions remain about the spatial and temporal dynamics regulating metal acquisition in the biofilm.

## METALS AND QUORUM SENSING

Quorum sensing is a sophisticated communication mechanism that enables bacteria to detect and respond to their population density through the production of small, diffusible signal molecules (135–137). This process is imperative in biofilms, where high cell densities and elevated nutrient demands impose coordinated group behavior. Metals, such as iron and manganese, can modulate quorum-sensing activity. These metals influence the production and reception of quorum-sensing signals, thereby affecting bacterial behaviors like biofilm formation, motility, and stress responses. Understanding the relationship between quorum sensing and metals is essential to identifying how bacteria adapt to their environments and manage communal activities like biofilms.

### P. aeruginosa

Quorum sensing in *P. aeruginosa* is a multitiered process controlled by four systems. Las and Rhl use N-acyl-homoserine lactones (AHL), whereas Pqs rely on quinolones and carbaldehyde signals (138). The LAS system responds to oxidative stress and activates the transcription of the gene encoding for manganese-cofactored superoxide dismutase (Mn-SOD) (139). During iron limitation in planktonic cells and biofilms, Mn-SOD activity increases, suggesting quorum-sensing activity (139). *P. aeruginosa* utilizes the Rhl quorum-sensing system to regulate rhamnolipid expression. Rhamnolipids are surface-active amphipathic molecules that function as a biosurfactant to reduce surface tension and increase twitching motility. Furthermore, rhamnolipids are critical for maintaining biofilm structure and contribute to biofilm dispersal (140–142). The Rhl system is induced in low-iron conditions, which stimulates twitching motility and abrogates biofilm production (Table 1) (83). The underlying mechanism for this switch is through rhamnolipid synthesis that results in a thin layer biofilm when increased in early biofilm formation in low iron (84). Mutants unable to generate rhamnolipids form strong biofilms and lack twitching motility (84). Furthermore, when *P. aeruginosa* is deficient in a siderophore called pyoverdine, quorum-sensing signaling is impacted for both the Las and Pqs systems (85). Overall, iron availability can modulate quorum-sensing activity, affecting bacterial behaviors such as biofilm formation, motility, and stress responses. This interplay between iron and quorum sensing highlights the complexity of bacterial communication and adaptation to their environment.

### Acinetobacter baumannii

A significant barrier to *A. baumannii* treatment is an evolved resistance to many antibiotics, including penicillins, cephalosporins, and carbapenems. *A. baumannii* forms biofilms on abiotic surfaces, like glass and plastic, which can be medically relevant for endotracheal tubes or intravascular catheters (65, 143). Growth of *A. baumannii* under iron limitation leads to a significant increase in biofilm development in comparison to cells grown in iron-replete conditions (65). Not only does *A. baumannii* encounter iron sequestration in the host, but it is expected that environmental surfaces are also iron-deficient. Therefore, biofilm formation could provide protection under these conditions. To understand if iron limitation impacts quorum sensing and biofilms, 65 multidrug-resistant *A. baumannii* strains were isolated from patients hospitalized in intensive care units (66). Under iron limitation, AHL and siderophore secretion were regulated by iron concentration (Table 1) (66). Isolates that produced high levels of AHL in low iron generated more biofilm, which could be reversed by adding iron back (66). Thus, *A. baumannii* coordinates biofilm formation under iron-limited conditions to improve survival and pathogenicity, particularly in medical environments. The regulation of biofilm formation through quorum-sensing molecules, such as AHL, emphasizes the adaptive strategies employed by this pathogen as it adjusts to environments outside of the host.

### Burkholderia cenocepacia

*Burkholderia cenocepacia* is a gram-negative bacterium that exists in the environment and is notorious for causing opportunistic lung infections in immunocompromised individuals, including people with cystic fibrosis (144). *B. cenocepacia* is one of at least 17 phenotypically similar species known as the Burkholderia cepacia complex, which accounts for approximately 45% of isolates from cystic fibrosis patients and is frequently associated with complicated disease and cepacia syndrome (145, 146). *B. cenocepacia* utilizes the Burkholderia diffusible signaling factor (BDSF) quorum-sensing system to regulate virulence and biological functions, like biofilm formation (26). *B. cenocepacia* utilizes the membrane-bound transporter MntH to acquire manganese, and MntH influences the BDSF quorum-sensing system, indicating that manganese uptake functions to support quorum sensing in *B. cenocepacia* (70). Furthermore, *B. cenocepacia* deficient in manganese uptake has decreased swarming motility and increased biofilm formation, suggesting that manganese controls the ability to form biofilms (Table 1) (70). Manganese uptake via MntH is crucial for regulating the BDSF quorum-sensing system and biofilm formation in *B. cenocepacia*, although the mechanisms remain unclear. Together, these findings suggest that manganese uptake via MntH is essential for coordinating quorum sensing and biofilm dynamics in *B. cenocepacia*, supporting a critical link between metal homeostasis and bacterial communication.

Metals, like iron and manganese, significantly influence quorum sensing, which is essential for bacterial communication and coordination. By modulating quorum sensing signals, these metals affect critical bacterial behaviors such as biofilm formation, motility, and stress responses. These studies enrich our understanding of quorum-sensing regulated responses during infection. Moreover, the mechanism by which metals regulate quorum sensing may be a conserved strategy across numerous bacteria and therefore may represent a new therapeutic target for the treatment of antimicrobial-resistant infections.

## SIDEROPHORES AND BIOFILMS

Iron mediates redox reactions in biological systems. In heme and iron-sulfur clusters, iron functions as an electron donor or acceptor (31). The availability of iron to bacteria is often a limiting factor in microbial growth, and bacteria utilize metal acquisition systems to obtain iron from their environment, including the production of siderophores (147–149). Siderophores have long been recognized as key virulence factors in bacterial pathogens, primarily due to their ability to bind to iron with a high affinity and outcompete host proteins, like calprotectin, lipocalin, transferrin, and lactoferrin. Calprotectin is a heterodimer of two S100 proteins, S100A8 and S100A9, that binds and sequesters zinc, manganese, iron, copper, cobalt, and nickel from pathogens (42, 150, 151). Calprotectin comprises up to 50% of the cytosolic protein content in neutrophils (152), and these innate immune cells are considered a primary source for this protein during infection. However, macrophages also produce calprotectin in response to infection (153). Calprotectin has antimicrobial activity against bacteria, like *S. aureus*, *A. baumannii, Clostridioides difficile, Y. pestis*, *Helicobacter pylori*, and *Mycobacterium tuberculosis* (43, 154–159). However, recent discoveries have revealed additional roles for siderophores, reshaping our understanding of their broader impact on bacterial pathogenesis.

### S. epidermidis

*S. epidermidis* requires iron for biofilm formation and utilizes a siderophore-mediated iron acquisition to form biofilms (160). Furthermore, the loss of iron homeostasis can severely impact biofilm formation (61). Catecholamines, predominantly epinephrine and norepinephrine, are used in intensive care medicine to maintain or stabilize blood pressure and improve cardiac function in hospital settings. *S. epidermidis* is stimulated by catecholamines to form biofilms on medically relevant materials (100). Several bacteria use host-derived catecholamines to chelate iron from host proteins during infection to make iron more readily available for siderophore binding (161–165). Moreover, *S. epidermidis* can specifically utilize catecholamine inotropes that are used as therapeutics in healthcare to remove iron from transferrin (101). Thus, *S. epidermidis* can take advantage of intravenous treatments to scavenge iron from host proteins, allowing siderophores to bind to iron and provide the nutrient metal to *S. epidermidis* to establish biofilms on catheters (Table 1).

### P. aeruginosa

*P. aeruginosa* produces a high-affinity and a low-affinity siderophore, called pyoverdine and pyochelin, respectively, that allow the bacteria to thrive in iron-limited environments to promote biofilm development (Table 1) (63, 166). *P. aeruginosa* forms biofilms, and in a wound model of infection, the genes for pyochelin synthesis are the only iron acquisition genes upregulated (86). Furthermore, pyochelin is isolated from the sputa of people with cystic fibrosis, indicating that *P. aeruginosa* experiences iron limitation (167). Without pyoverdine, *P. aeruginosa* is unable to develop biofilms regardless of iron availability in the environment, and biofilm structure is modified (63, 85). In the context of biofilm architecture, pyoverdine biosynthetic enzymes are dramatically upregulated at the edge of the biofilm during iron limitation (29). Accordingly, induction of pyoverdine synthesis at the edge of biofilm formation is reversed with the addition of zinc (29). Moreover, pyoverdine can bind to zinc; bacteria lacking pyoverdine are attenuated in zinc limitation, and pyoverdine production is enhanced by zinc (166, 168–171). These data suggest that pyoverdine synthesis can be impacted by zinc and pyoverdine can transport zinc, indicating that zinc limitation also impacts biofilm dynamics by pyoverdine, although this has not been directly tested.

*P. aeruginosa* is one of the most common bacteria to infect the cornea to cause keratitis, which can lead to complete destruction of the cornea in 2 days (172, 173). In corneal tissues from individuals with keratitis, S100A12, also known as calgranulin, has elevated expression (92). Similar to calprotectin, calgranulin can be found in neutrophils and is a zinc and copper-binding protein (174, 175). In the presence of calgranulin, *P. aeruginosa* decreases the expression of pyoverdine synthesis and secretion (92). Furthermore, calgranulin exposure decreases transcription of biofilm genes, such as those involved in quorum sensing, lectins, and extracellular matrix, and thus reduces biofilm formation (92). Expression of calgranulin is increased in lung biopsies from people with cystic fibrosis (176). These findings suggest that calgranulin plays a significant role in modulating bacterial behavior and biofilm formation in response to zinc availability. Identification of the interaction between calgranulin and *P. aeruginosa* could provide new insights into therapeutic strategies for managing infections in vulnerable tissues like the cornea and lungs. The impact of calgranulin on bacterial pathogenesis is an under-explored area, and further research is needed to discover potential calgranulin pathways that could be targeted to control biofilm-related infections and improve patient outcomes.

### E. coli

Urinary tract infections are the most prevalent infections among individuals with indwelling urinary catheters. A significant challenge in these infections is the formation of bacterial biofilms. The majority of urinary tract infections are caused by the gram-negative bacterium *E. coli* (177). The concentration of iron in the urinary tract increases during urinary tract infection (178). Furthermore, a gene cluster on a pathogenicity island encoding for the yersiniabactin siderophore system was shown to be upregulated in the urinary tract *in vivo* (77). Moreover, a gene encoding a putative yersiniabactin (pesticin) receptor protein homologous to *fyuA* was one of the most upregulated genes grown in the biofilm in urine (Table 1) (77). Moreover, *E. coli* lacking *fyuA* is deficient in biofilm growth in static and constant flow conditions, similar to those found on a catheter (77). Therefore, *E. coli* utilizes the yersiniabactin system to acquire iron in the urinary tract and enable robust biofilm formation on catheters to contribute to the persistence of catheter-associated urinary tract infections.

### K. pneumoniae

A large portion of iron in the human body is stored in the liver (179). The liver’s iron-rich environment provides an ideal niche for certain *K. pneumoniae* strains to establish infection and form biofilms that contribute to liver abscess development (Table 1) (79). Some strains of *K. pneumoniae* take advantage of these high iron concentrations to infect the liver and form liver abscesses. High iron concentrations lead to biofilm formation by *K. pneumoniae,* and increasing iron levels enhance biofilm formation, but iron chelation attenuates biofilms (79, 80). Furthermore, *K. pneumoniae* strains that can cause liver abscesses secrete four siderophores, aerobactin, yersiniabactin, salmochelin, and enterobactin, whereas strains that do not cause abscesses secrete only enterobactin (79). Genes encoding siderophore systems in abscess-forming bacteria are downregulated in high iron conditions. On the contrary, enterobactin genes in non-abscess-forming bacteria are increased in high iron concentrations compared with limited iron (79). The ability of *K. pneumoniae* strains to produce multiple siderophores has been linked with hypervirulence and hypermucoviscosity, which enhances liver abscess formation (180, 181). Strains capable of producing multiple siderophores exhibit enhanced virulence and hypermucoviscosity that allow them to thrive in high-iron conditions and evade host defenses.

### Burkholderia cenocepacia

Protein glycosylation is increasingly recognized as a common post-translational protein modification in bacterial species. *B. cenocepacia* utilizes an O-linked glycosylation system responsible for the modification of at least 23 proteins (182). In *B. cenocepacia*, CepR functions as a quorum-sensing receptor for biofilm formation and swarming motility (183). Loss of glycosylation in *B. cenocepacia* represses CepR to alter the global proteome beyond the known glycoproteome, including changes in biofilm and siderophore activity (69). A glycosylation enzyme mutant is deficient in biofilm formation and ferric iron-siderophore secretion (Table 1) (69), highlighting a correlation between bacterial communication and shared resources.

In the soil, *Bacillus subtilis* uses the siderophore bacillibactin for iron uptake and biofilm formation (Table 1) (68). *B. subtilis* can cause septicemia, endocarditis, and pneumonia, but typically only in immunocompromised individuals. The *B. subtilis* biofilm structure not only promotes iron homeostasis but also promotes siderophore-dependent iron acquisition (68). Given that bacteria in a dense community can share nutrients and common goods more efficiently than those spread out in an environment, it is not surprising that there is a clear link between the regulation of siderophore production and biofilm formation for bacteria mentioned above and others like *P. aeruginosa*, *B. cenocepacia, E. coli, Y. pseudotuberculosis*, *Mycobacterium smegmatis*, *Legionella pneumophila*, and *Cupriavidus necator* (77, 107, 119, 184–186). Furthermore, many pathogens can produce multiple siderophores, a trait initially believed to serve as a form of functional redundancy. However, emerging evidence suggests that different siderophores within a single pathogen may confer specific advantages depending on the infection’s location and nature. *A. baumannii* produces up to 10 siderophores; however, only acinetobactin has been found to be essential for virulence in serum and host tissues, indicating that different siderophores serve distinct roles depending on the infection niche (187). Similarly, *K. pneumoniae* secretes aerobactin, enterobactin, salmochelin, and yersiniabactin, each contributing uniquely to pathogenesis, immune evasion, and dissemination, with salmochelin, aerobactin, and yersiniabactin evading host sequestration by lipocalin-2 and yersiniabactin also binding copper to resist metal intoxication (180, 188). With recent findings of metallophore promiscuity and functionality, key questions remain regarding how pathogens regulate siderophore expression, how these molecules interact with non-iron metals, and how host defenses respond to their diversity.

## POLYMICROBIAL BIOFILM METAL INTERACTIONS

### E. faecalis

*E. faecalis* is frequently associated with infections of the urinary tract, catheters, and surgical wound sites. These catheter-associated and wound infections are often polymicrobial with frequently co-isolated bacteria including *E. coli, S. aureus*, *A. baumannii, Klebsiella* species, *P. aeruginosa*, and *Proteus mirabilis* (189–193). There is an urgent need to identify new therapeutics for polymicrobial biofilms because they are often associated with higher mortality rates, increased hospital and intensive care stays, and increased healthcare costs than biofilms with a singular organism. *E. faecalis* promotes the growth and survival of *E. coli* when iron is limited (127). This interaction occurs during biofilm formation and is dependent on the production of the siderophore enterobactin (Table 1) (127). *E. faecalis* can apply mechanisms to hinder the growth of other resident microbes in the biofilm. *P. aeruginosa* is often an antagonist that produces antimicrobial compounds to outcompete other bacteria (194). In iron-restricted conditions, *E. faecalis* acidifies the environment by producing lactic acid, leading to L-lactate formation and a reduction in pH, which eventually exceeds a pH threshold at which *P. aeruginosa* can grow (74). The L-lactate in the environment also chelates iron in the media away from *P. aeruginosa* (Table 1) (74). Overall, these iron-dependent mechanisms allow *E. faecalis* to withstand antimicrobial strikes from *P. aeruginosa* and survive in the biofilm environment.

### P. aeruginosa

In people with cystic fibrosis, the presence of *P. aeruginosa* is often linked to a reduced possibility of co-infection with other common pathogens, such as *S. aureus*, Burkholderia cepacia complex, *Stenotrophomonas maltophilia*, and *Achromobacter xylosoxidans* (195). Over time, once *P. aeruginosa* establishes itself in the airway, its population tends to increase, gradually becoming the dominant species as the prevalence of other microbes declines (196). Respiratory viral infections can increase iron availability in the lungs, which enhances the ability of *P. aeruginosa* to form biofilms and outcompete other bacteria (197). This competitive edge is linked to the expression of the type VI secretion system and a specific toxic protein, TseT, regulated by the Las quorum-sensing system and triggered by various iron sources, such as transferrin and hemoglobin (Table 1) (87). This mechanism affects interactions with other common cystic fibrosis pathogens, such as *A. xylosoxidans* and *S. maltophilia*, and is observed both in lab models and clinical settings (87).

*S. aureus* can often co-exist with *P. aeruginosa* in cystic fibrosis airways, despite *P. aeruginosa* generating anti-bacterial compounds (198, 199). Consequently, increased polymicrobial infections in people with cystic fibrosis lead to an increase in inflammation and release of calprotectin (200). Since calprotectin is a metal-sequestering protein, this suggests a metal-limiting environment for bacterial pathogens. However, the presence of calprotectin supports *P. aeruginosa* and *S. aureus* coinfections in the lung (Table 1) (29). This emerges by metal limitation repressing biosynthetic genes responsible for the production of numerous anti-staphylococcal factors in *P. aeruginosa* (29, 93, 94). Calprotectin has also been shown to have other antimicrobial properties outside of metal sequestration through direct contact with the bacterial surface (153). Correspondingly, *P. aeruginosa* and *S. aureus* biofilm communities in monoculture and coculture become encapsulated by calprotectin (201). Biofilm formation by *S. aureus* was induced in the presence of calprotectin, suggesting a mechanism where host proteins can impact biofilm development. Furthermore, the composition of the carbohydrates in the extracellular polymeric substance of the *P. aeruginosa* biofilm was altered by calprotectin binding (201). Moreover, *S. aureus* can convert a siderophore produced by *P. aeruginosa,* pyochelin, to an analog with a reduced affinity for iron (99). These mechanisms promote increased tolerance between *S. aureus* and *P. aeruginosa* in the biofilm. They also reveal how metals can impact the formation of robust dual-species biofilms.

*P. aeruginosa* not only colonizes biofilms with bacteria but also with fungi like *Candida albicans and Aspergillus fumigatus*. These opportunistic pathogens form biofilms in the lungs of people with cystic fibrosis (202). *C. albicans* is a polymorphic fungus, and *P. aeruginosa* binds to *C. albicans* filaments to generate a polymicrobial biofilm and kills the fungi but cannot bind to the yeast form (95). In this environment, *P. aeruginosa* upregulates genes encoding for siderophore systems, and analogously, pyoverdine production is increased in mixed biofilms (Table 1) (96, 97). These data suggest that *C. albicans* is under iron-limited conditions in the biofilm environment. However, other studies show that induction of iron and zinc acquisition pathways in *C. albicans* is not increased in polymicrobial compared with monomicrobial biofilms (98). Variations in these findings could be explained by differences in *in vitro* growth conditions. Interestingly, zinc limitation is linked to a hyper-adhesion phenotype across pathogenic *Candida* species (203), indicating that metal limitation could impact early biofilm development. Thus, although it is unclear if *P. aeruginosa* kills *C. albicans* through metal limitation, it is quite possible that the development of this polymicrobial biofilm arises due to the zinc-restricting conditions in the cystic fibrosis airway.

In the multi-kingdom *P. aeruginosa* and *A. fumigatus* biofilm, *P. aeruginosa* inhibits the growth of the fungus in an iron-dependent mechanism (Table 1) (88, 89). *A. fumigatus* increases the expression of genes responsive to iron starvation when bacteria are present (89). Moreover, a reduction in pyoverdine production by *P. aeruginosa* decreases antifungal activity against *A. fumigatus* (89). *P. aeruginosa* isolates from the lungs of 10 people with cystic fibrosis had antifungal properties against an *A. fumigatus* biofilm (89). Furthermore, a correlation arose between the concentration of pyoverdine in patient samples and the ability to inhibit fungal biofilms, validating an iron-dependent mechanism for inhibition (89). These interactions emphasize the importance of metal-dependent mechanisms in shaping biofilm dynamics and influencing the pathogenicity of opportunistic organisms, especially in environments like the cystic fibrosis airway. Further research is needed to fully explore how bacterial species in mixed biofilms compete or cooperate to acquire metals in polymicrobial settings.

## EXAMPLES OF THE IMPACT OF NUTRITIONAL IMMUNITY ON CLINICAL BIOFILMS

### Cystic fibrosis

People with cystic fibrosis experience increased labile iron levels in the lung microenvironment in comparison to individuals without cystic fibrosis due to acidification of the airway (204–208). This is further complicated by reports that people with cystic fibrosis have increased iron deficiency, which is attributed to diminished iron absorption in tissues and increased iron released from the body through sputum (209, 210). Meanwhile, the iron-rich environment in the lung provides ideal conditions for bacterial growth and infection for opportunistic pathogens like *P. aeruginosa* and *B. cenocepacia* (205, 211). High iron concentrations cause aggregation of bacterial cells, adhesion, and biofilm formation for both *P. aeruginosa* and *B. cenocepacia* (206, 211). In fact, a combination of tobramycin, an antibiotic commonly used to treat bacterial infections in people with cystic fibrosis, with an iron chelator significantly reduces established *P. aeruginosa* biofilm biomass and decreases the number of viable bacteria, further validating iron’s stimulatory role in biofilm establishment (212). People with cystic fibrosis widely use the effective CFTR modulator therapy elexacaftor/tezacaftor/ivacaftor (ETI) that significantly lowers the bacterial load in the lungs (213). In a study conducted with *P. aeruginosa* sequences isolated from people with cystic fibrosis pre- and post-ETI therapy, the types of gene mutations in *P. aeruginosa* changed in four out of six participants (214). Specifically, there was a significant shift in mutations affecting genes related to iron acquisition through siderophores like pyoverdine and pyochelin. This suggests that the respiratory environment post-ETI alters selective pressures on bacterial iron acquisition strategies.

*P. aeruginosa* uses an exopolysaccharide called Psl in the biofilm matrix that is enhanced in high iron conditions (Table 1) (90). The bacterium utilizes Psl as an iron storage framework, where ferrous and ferric iron can be interwoven into the biofilm (90). Additionally, iron bound to Psl can be exploited by *P. aeruginosa* as a nutrient resource (90). Clinical isolates of *P. aeruginosa* often express a different phenotype showing an increase in the polysaccharide alginate and mucoid biofilm in the presence of iron limitation (91). Chronic colonization in people with cystic fibrosis is linked to a mucoid biofilm phenotype that is difficult to eliminate (215, 216). The iron-rich environment of the cystic fibrosis lung and disrupted iron homeostasis creates ideal conditions for persistent bacterial infections. Pathogens like *P. aeruginosa* capitalize on this by integrating iron into their biofilm matrix to enhance survival and resistance to treatment.

In clinical isolates of sputa from individuals with cystic fibrosis infected with *P. aeruginosa*, lactoferrin is found at high concentrations (217). Lactoferrin is a member of the transferrin family and is found within the specific granules of neutrophils and in biological fluids across mucosal sites (218, 219). Lactoferrin serves as a host antimicrobial protein by binding to the surface of gram-negative bacteria, binding lipopolysaccharide, and sequestering iron from bacteria (44, 220, 221). Furthermore, lactoferrin blocks biofilm formation of *P. aeruginosa* by chelating iron (Table 1) (63). Similarly, *P. aeruginosa* derived from chronic wound biofilms is attenuated in the presence of lactoferrin, suggesting that iron starvation impacts biofilms in diabetic ulcers and cardiovascular disease (14, 222). People with cystic fibrosis who are colonized with *P. aeruginosa* have increased levels of the protease cathepsin released from resident immune cells, which cleaves lactoferrin and alleviates barriers for biofilm retention in the lung (223). The elevated presence of cathepsin degrades lactoferrin to undermine this host defense mechanism and ultimately facilitate persistence of biofilm-associated infections in the cystic fibrosis lung.

### Diabetic wounds

Chronic wounds are a common and serious complication of diabetes, affecting approximately 25% of individuals with the condition (224). These wounds, such as diabetic ulcers, are frequently exacerbated by infections involving biofilms, which contribute to their persistence and resistance to treatment (14, 225, 226). In diabetic mice, despite the presence of calprotectin and lipocalin-2, a siderophore-binding protein, Group B Streptococcus (GBS) is not restricted in a wound model, indicating a failure of the host’s nutritional immunity (46). In contrast, non-diabetic wounds rely on bacterial metal transporters for GBS survival, suggesting that metal sequestration by the immune system is more effective in these environments (46). This disparity implies that either the immune system in diabetic wounds is less capable of sequestering nutrient metals or that metals are more readily available. Ultimately, this altered metal homeostasis in diabetic wounds undermines the host’s ability to control GBS infections, promoting bacterial persistence and potentially enhancing biofilm formation.

### Stomach ulcers

*H. pylori* is a gram-negative bacterium that colonizes about half of the world’s population. Colonization with *H. pylori* can lead to peptic ulcer disease and the development of stomach cancer. Thus, the World Health Organization has classified *H. pylori* as a class I carcinogen. *H. pylori* triggers an inflammatory response with infiltration of immune cells into the lamina propria and gastric mucosa, leading to a large release of calprotectin. The metal-limited conditions induced by calprotectin enhance *H. pylori* biofilm formation (Table 1) (78). The alteration in lifestyle is caused by a disruption in the synthesis of lipid A (78). Antibiotic treatment can reduce the risk of gastric cancer development; however, biofilm formation is linked to an increase in antibiotic efflux pump expression and resistance mutations (227, 228).

Nutritional immunity is an understudied aspect in microbial biofilm formation. With the persistence of biofilms in the clinical setting, new treatments are needed to reduce and manage the occurrence of biofilm formation. It is valuable for us to gain an understanding of how our immune system succeeds and fails at limiting biofilms and if we can exploit those processes for preventative responses.

## METAL-DEPENDENT THERAPEUTICS FOR BIOFILMS

Specific metals show the ability to prevent biofilm formation in culture. The ability to form and stabilize biofilms was tested with three well-studied biofilm formers, *E. coli*, *S. aureus*, and *P. aeruginosa,* with increasing concentrations of metal salts to determine which metals have anti-biofilm properties and compared with the inhibition of planktonic growth (Table 2). Copper, silver, titanium, gallium, and aluminum prevented biofilm formation of both *E. coli* and *P. aeruginosa* (229). Also, zinc prevents the formation of *S. aureus* and *E. coli* (229). A more important aspect of a candidate’s therapy is the eradication of the established biofilms. Thus, when metals were tested on formed biofilms, copper, silver, titanium, gallium, and aluminum could remove biofilms for all three bacteria (229). Nickel was able to prevent *S. aureus* biofilm formation (229). *A. baumannii* biofilms are also susceptible to copper treatment (230). Although specific metals repetitively showed antimicrobial activity, there was a large variation in concentrations and exposure times, indicating that no one metal will likely be the solution for treating biofilms. It is much more likely that metals can be used in combination with other therapeutics to increase efficacy (231, 232).

**TABLE 2:** Metals with antibiofilm activity

Metal	Bacteria	References
Aluminum	*E. coli*, *P. aeruginosa*, *S. aureus*	(229)
Copper	*E. coli, P. aeruginosa*, *S. aureus, A. baumannii*	(229, 230)
Gallium	*E. coli, P. aeruginosa*, *S. aureus*	(229)
Iron	*S. epidermis*, *A. baumannii, S. aureus, K. pneumoniae*	(80, 229)
Nickel	*E. coli, S. aureus*	(229)
Silver	*E. coli, P. aeruginosa*, *S. aureus*	(229)
Titanium	*E. coli, P. aeruginosa*, *S. aureus*	(229)
Zinc	*E. coli, S. aureus*	(229, 233)

Iron can work in combination with antibiotics and bacteriophages to deplete biofilms. The urinary antibiotic nitroxiline and compound HP-14 have iron-chelating effects on *P. aeruginosa*, *S. epidermidis*, *A. baumannii,* and methicillin-resistant *S. aureus* (MRSA) (234, 235). Moreover, HP-14 destroys MRSA biofilms and eradicates persister cells (236, 237). In a population, persister cells represent metabolically dormant cells that do not respond to antibiotic treatment and become active post-antibiotics. This population will begin to divide and reform biofilms (238). Thus, this population is typically difficult to eliminate. Furthermore, using iron chelation in combination with a bacteriophage reduces *K. pneumoniae* biofilms (80). Bacteriophages infect bacteria to hijack the cell; thus, these bacteria-specific viruses can pose advantages over antibiotics, such as specificity and self-replication (239, 240). Furthermore, specific phages have been identified to degrade biofilm matrix to trigger disruption and support antimicrobial access into the milieu (241). By disrupting metal availability, compounds like nitroxoline and HP-14 not only impair biofilm integrity but also target difficult-to-treat persister cells, whereas bacteriophages offer precision and matrix-degrading capabilities that further support biofilm eradication.

Zinc as an antimicrobial compound represents a promising therapeutic at high concentrations as it is inexpensive, easily accessible, and has minimal effects on the human body. Zinc sulfate and zinc chloride present antibiofilm properties. In one study, 50 isolates of *S. aureus* were obtained from patients with surgical or burn-infected wounds, with around 68% of the isolates producing biofilms (233). Zinc sulfate demonstrated anti-biofilm activity against *S. aureus* by inhibition of biofilm formation for all isolates and downregulating biofilm genes, like *icaA*, *icaD*, *icaB*, and *fnb*A (233). Importantly, zinc sulfate showed a synergistic effect against planktonic cells with the majority of antibiotics tested, suggesting that treatment together with these compounds could disperse biofilms and eliminate bacterial colonization (233). Zinc demonstrates strong potential as an accessible and cost-effective antimicrobial agent, particularly through its ability to inhibit biofilm formation and enhance antibiotic efficacy.

Synthetic lactoferrin has bactericidal properties against multidrug-resistant *A. baumannii* (242). In the context of biofilms, lactoferrin can penetrate the interior of some biofilms, which could support antimicrobial compounds entering the matrix (222). This activity can be seen in *S. epidermidis* biofilms, where, in combination with vancomycin, lactoferrin decreased biofilms (243). Lactoferrin has been shown to be synergistically efficacious as an anti-biofilm therapy with xylitol, which is a sugar alcohol taken in by bacteria that cannot be metabolized (244, 245). In addition to its growth-inhibitory effect for bacteria, lactoferrin can inhibit bacterial adhesion. Lactoferrin impacts the growth of *S. mutans* in an iron-independent manner (221). This is similar to pathogenic *E. coli*, where lactoferrin blocks adhesion of the bacteria (246). In *S. pneumoniae* biofilms, lactoferrin reduces biofilms by eliminating DNA from the matrix (247). Although the mechanisms for lactoferrin-mediated biofilm inhibition are diverse, the host protein limits biofilm formation. New approaches are emerging as attempts to prevent or reduce bacterial infections, such as blocking adhesion of pathogens to the surface of the host cell, where lactoferrin may be a potential therapeutic.

The application of metals such as copper, silver, titanium, gallium, and aluminum as therapeutics has great promise in preventing and eradicating biofilm infections. These metals can disrupt biofilm formation and enhance the effectiveness of traditional antibiotics and even new treatments. Zinc demonstrates anti-biofilm properties and works synergistically with antibiotics to disperse biofilms and eliminate bacterial colonization. By incorporating these metal treatments into existing therapeutic strategies, we can improve our ability to combat persistent biofilm infections and enhance patient outcomes.

## CONCLUSION

In conclusion, metals shape biofilm dynamics and bacterial pathogenesis beyond nutritional demand. By influencing biofilm architecture, resilience, and microbial interactions, metals significantly impact bacterial virulence, the ability to evade host defenses, and resistance to antimicrobial therapeutics. By defining these critical interactions, we can identify opportunities for therapeutic intervention and offer potential strategies to disrupt biofilm formation and eventually prevent biofilm-associated infections. Future research should continue to explore the interplay between metals and biofilms, as several key questions remain that are critical for understanding biofilm development and biofilm-associated infections.
